# Renal Outcome in Patients Undergoing Cardiac Surgery Using Cardiopulmonary Bypass

**DOI:** 10.7759/cureus.9015

**Published:** 2020-07-06

**Authors:** Mohammed S Alqarni, Abdullah H Ghunaim, Abdulkarim W Abukhodair, Jose Andres Fernandez, Sean R Bennett

**Affiliations:** 1 Medicine, King Saud Bin Abdulaziz University for Health Sciences, Jeddah, SAU; 2 Medicine, College of Medicine, King Abdulaziz University, Jeddah, SAU; 3 Anesthesiology, King Faisal Cardiac Center, King Abdullah Medical City, Jeddah, SAU

**Keywords:** renal outcome, cardiac surgery, cardiopulmonary bypass, kingdom of saudi arabia (ksa)

## Abstract

Introduction

Renal dysfunction is a significant variable in determining the outcome of surgery, such as cardiopulmonary bypass graft and valvular replacement, used to treat cardiovascular diseases. In Saudi Arabia, the incidence of renal failure and diabetes is higher than in most western populations. Our aim is to determine the renal outcome of patients who underwent cardiac surgery at King Faisal Cardiac Center from 2014 to 2017.

Methods

This a retrospective cohort study using a non-probability consecutive sampling technique for selection of the study population to assess the renal outcome in cardiac surgery patients using cardiopulmonary bypass from May 2014 to June 2017 in King Faisal Cardiac Center, Jeddah. Patients older than 18 years of age undergoing cardiac surgery, with available data, were included. Categorical variables were summarized by percentages and frequencies, and continuous variables by means and standard deviations, or medians and interquartile ranges if their distributions were skewed. Logistic regression was done with post-op renal impairment as the dependent variable and pre-op renal dysfunction, age, gender, smoking status, diabetes, hypertension, dyslipidemia, and cardiopulmonary bypass time as independent variables.

Results

Our sample size included 244 patients who underwent cardiac surgery in this study period; their mean age was 60.5 (SD =7.5) with a mean body mass index (BMI) of 28.62 (SD=5.19). Among our population, 73% (n = 179) were males and 27% (n =66) were females. Two percent (2%) of patients (n = 5) died within 30 days, 4% of patients (n = 10) with temporary dialysis, 8% of patients (n = 19) with postoperative renal dysfunction, and no patients with permanent dialysis. The data showed a significant relationship between levels of creatinine preoperatively and postoperative renal dysfunction (p-value = 0.0001, OR=1.05, 95% CI of 1.031 to 1.064).

Conclusion

The main predictor of poor renal outcomes for cardiac surgery is preoperative creatinine. While other factors, such as age, gender, body mass index, cardiopulmonary bypass time, diabetes, hypertension, and dyslipidemia, did not show any risk to the postoperative renal outcome.

## Introduction

According to the World Health Organization (WHO), in 2017, more than 31% of the deaths in the world (17.9 million people) were due to cardiovascular disease [1]. Cardiovascular diseases are common in the population due to the increased amount of modifiable risk factors, such as high blood pressure, dyslipidemia, smoking, diabetes mellitus, obesity, and chronic kidney disease (CKD), and non-modifiable risk factors such as age, family history and gender [2-3]. These risk factors increase the risk of getting these diseases and could affect severity [4-5].

Renal dysfunction is also a major risk factor for end-stage renal failure and premature death [6]. A paper in 2010 calculated the global prevalence of CKD to be around 497 million adults in the world, which clearly proves that this is a global problem [7]. In addition, according to the Global Burden of Disease Study 2013, there was a 134.6% increase in the mortality of CKD patients since 1990, with a staggering 956,200 deaths in 2013 alone [8].

Renal dysfunction is a significant variable in determining the outcome of the surgeries used to treat these cardiovascular diseases - the most common surgeries being cardiopulmonary bypass graft (CABG) and valvular replacement (VR) [9]. Up to 30% mortality has been reported [10]. According to the literature, the severity of renal dysfunction also affects mortality [10-11]. In Saudi Arabia, the incidence of renal failure and diabetes is higher than in most western populations [12-13]. The King Faisal Cardiac Center (KFCC) at the National Guard Hospital Jeddah recently started cardiac surgery. Our aim is to determine the renal outcome of patients who underwent cardiac surgery at KFCC from 2014 to 2017.

## Materials and methods

This a retrospective cohort study using a non-probability consecutive sampling technique for the selection of the study population to assess the renal outcome in cardiac surgery patients using cardiopulmonary bypass from May 2014 to June 2017 in KFCC, Jeddah. The research project was approved by King Abdullah International Medical Research Center, Jeddah, Saudi Arabia (KAIMRC) and the Institutional Review Board (IRB). Our data was collected from hard files and soft files (BestCare, ezCaretech, Jung-gu, Seoul) at medical records. Patients older than 18 years of age who were undergoing cardiac surgery with available data were included. The data collection sheet included the following demographics: age, gender, body mass index (BMI), type of cardiac surgery, co-morbidities such as diabetes mellitus, hypertension, and hyperlipemia. Also, preoperative serum creatinine levels, preoperative renal dysfunction, and cardiopulmonary bypass (CPB) time were also included as independent variables. The main outcomes were 30-day mortality, new dialysis, and postoperative renal dysfunction. Acute renal dysfunction is defined as serum creatinine levels greater than or equal to 200 μmol/L [14].

Categorical variables were summarized by percentages and frequencies, and continuous variables by means and standard deviations or medians and interquartile ranges if their distributions were skewed. Baseline univariate comparisons between postoperative renal dysfunction and no postoperative renal dysfunction were made with Wilcoxon test rank-sum and the chi-square tests where appropriate. Logistic regression was done with postop renal dysfunction as the dependent variable and preop renal dysfunction, age, gender, smoking status, diabetes, hypertension, dyslipidemia, and CPB time as independent variables. Independent variables with p-value <0.05 were considered significant. All results were computed using IBM SPSS version 23 (IBM Corp., Armonk, NY).

## Results

**Figure 1:**
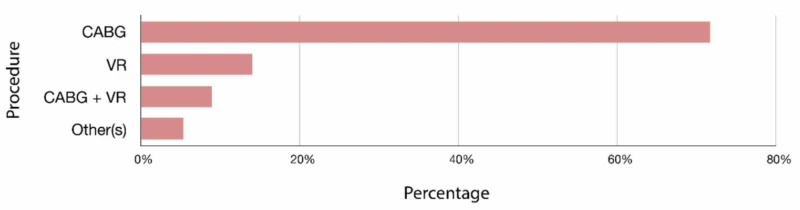
Types of procedures CABG: Coronary Arteries Bypass Graft, VR: Valvular Replacement

Our sample size included 244 patients who underwent cardiac surgery in this study period; their mean age was 60.5 (SD =7.5) with a mean BMI of 28.62 (SD=5.19). Among our population, 73% (n = 179) were males and 27% (n =66) were females. Co-morbidities included hypertension 80% (n = 197), diabetes 77% (n = 190), dyslipidemia 64% (n = 156), and smoking 27% (n = 65). The patients in this sample displayed a median 77 μmol/L (IQR = 66 - 99.75) of postoperative creatinine and a median of 117 minutes (IQR = 84-143) for bypass time. All the characteristics of the patients are summarized in Table 1 and Figure 1.

**Table 1:** Patients' characteristics BMI: Body Mass Index, CABG: Coronary Arteries Bypass Graft, VR: Valvular Replacement, CPB: Cardiopulmonary Bypass, S.D: Standard Deviation, IQR: Interquartile Range

Patient characteristic		n=244patients
Preoperative characteristics		
Age (years)		
Mean ± S.D.		60.5 ± 7.5
Male gender %		73%
BMI (kg/m^2^)		
Mean ± S.D.		28.62 ± 5.19
Smoking %		27%
Hypertension %		80%
Diabetes mellitus %		77%
Dyslipidemia %		64%
Preop creatinine (µmol/L)		
Median ± IQR		83 ± 31
Intraoperative characteristics		
Type of surgery (%)		
CABG		73%
Valve		17%
CABG + Valve		8%
Other		2%
CPB time in minutes		
Median ± IQR		117 ± 59
Postoperative characteristics		
Postop creatinine (µmol/L)		
Median ± IQR		77 ± 34
Temporary dialysis % (n)		4% (10)
Postoperative renal dysfunction % (n)		8% (19)
Mortality % (n)		2% (5)

**Figure 2:**
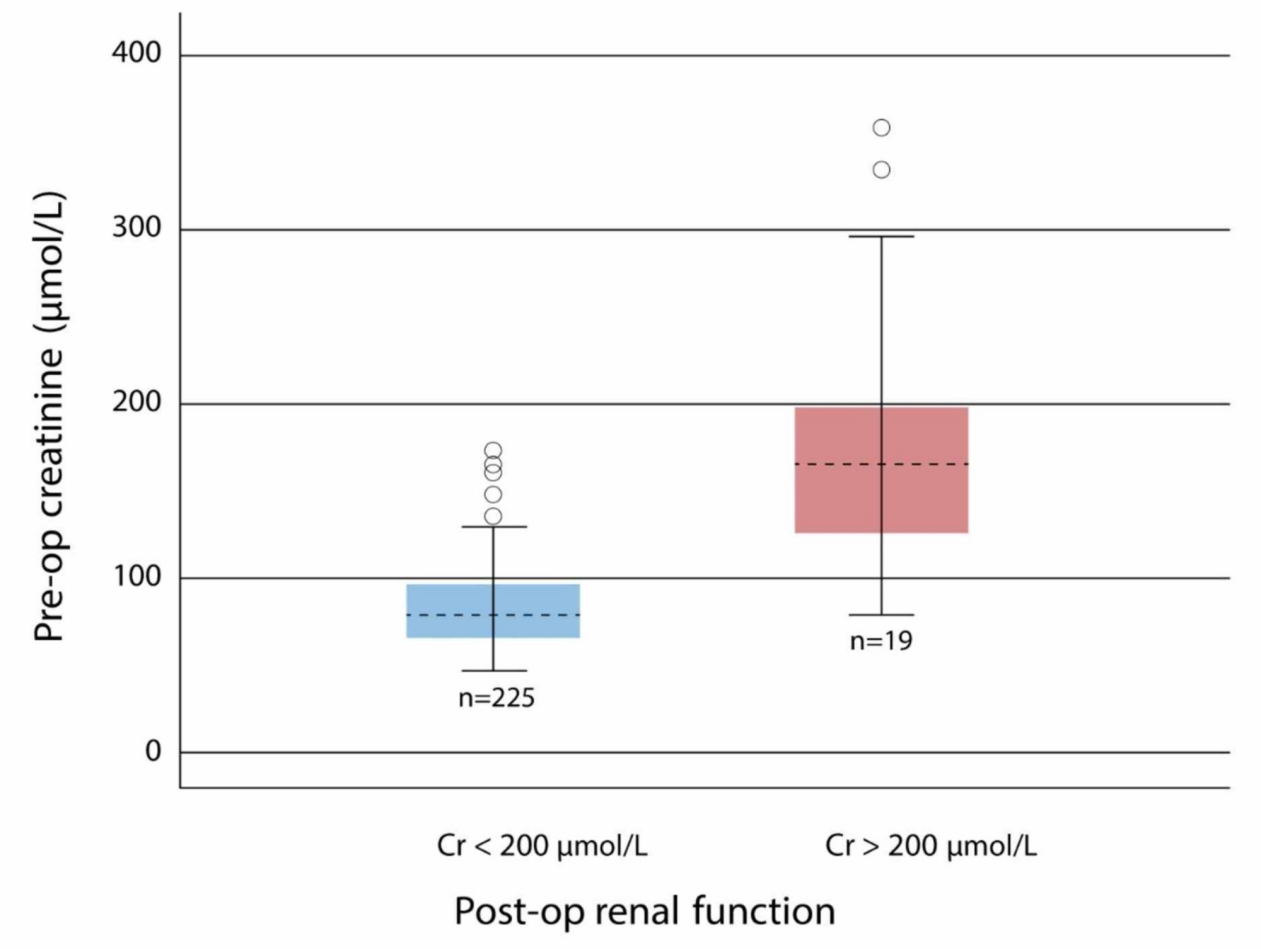
Preoperative creatinine in patients with postoperative renal dysfunction vs patients with no postoperative renal dysfunction

There were 2% of patients (n = 5) who died within 30 days, 4% of patients (n = 10) with temporary dialysis, 8% of patients (n = 19) with postoperative renal dysfunction, and no patients with permanent dialysis. Twenty-five patients had postoperative renal dysfunction, 19 of these had preop renal dysfunction. The data showed a significant relationship between the levels of creatinine preoperatively and postoperative renal dysfunction (p-value = 0.0001, OR=1.05, 95% CI of 1.031 to 1.064), as shown in Figure2.

**Figure 3:**
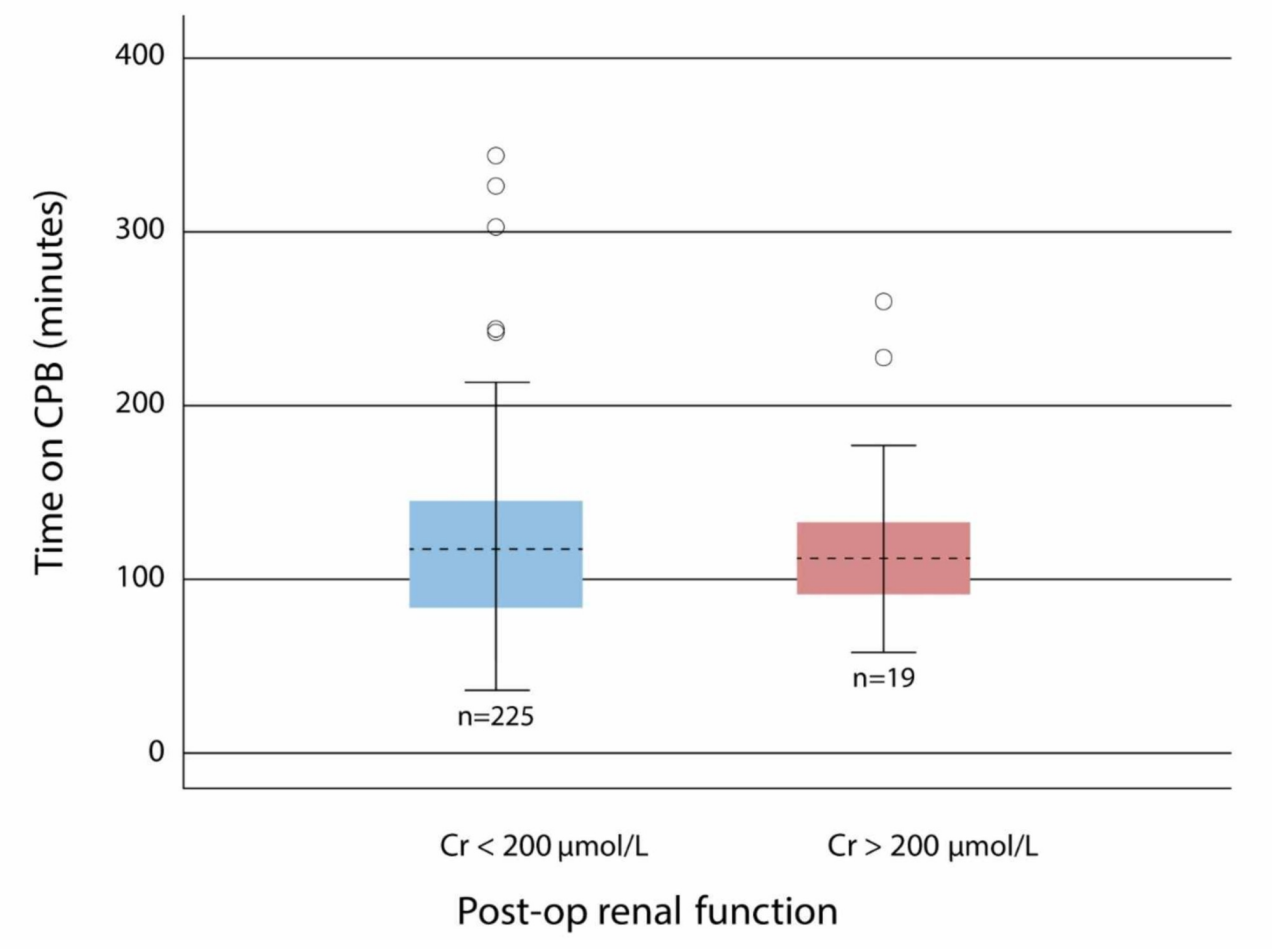
Time on CPB in patients with postoperative renal dysfunction vs patients with no postoperative renal dysfunction CPB: cardiopulmonary bypass

CPB time (p-value = 0.788), hypertension (p-value = 0.619), diabetes (p-value =0.961), and age (p-value = 0.180) were not statistically significant difference (Figure 3 and Table 2).

**Table 2:** Independent predictors of postoperative renal dysfunction B: Base, S.E.: Standard Error, S.D.: Standard Deviation, Wald: Wald Chi-Squared Test, df: degrees of freedom, C.I.: Confidence Interval, Exp: Natural Exponential Function

	B	S.E.	Wald	df	Significance	Exp(B)	Lower C.I. for EXP(B)	Upper C.I. for EXP(B)	
Creatinine	.048	.009	26.823	1	.001	1.050	1.031	1.069	
Time on bypass	-.002	.007	.072	1	.788	.998	.984	1.012	
Hypertension	.678	1.363	.247	1	.619	1.969	.136	28.458	
Age	.050	.038	1.801	1	.180	1.052	.977	1.132	
Diabetes	.060	1.228	.002	1	.961	1.061	.096	11.784	

## Discussion

CPB time has been associated with postoperative renal dysfunction in recent studies; however, our study did not show any statistical significance [15-16]. We suspect that the low sample size was the main reason that we got an insignificant association between postoperative renal dysfunction and CPB time. Our study has shown a significant association with preoperative renal function and postoperative renal dysfunction, and this is similar to many studies that show that preoperative renal dysfunction, as measured by serum creatinine levels, increases the risk of developing acute kidney injury [17-18].

Mortality after surgery, as well as postoperative temporary dialysis, was low. Also, none of the participants required permanent dialysis, and only 8% of our population showed postoperative renal dysfunction. In contrast to the other articles that showed a higher incidence of mortality, dialysis, and renal dysfunction [16-18].

There were more males compared to females, similar to the results from different articles that have been conducted with a male-to-female ratio of almost 3:1 [16-19]. Demographic data and cardiac co-morbidities, such as hypertension, diabetes, and dyslipidemia, did not show any statistically significant association with postoperative renal dysfunction. Likewise, a study that was done in Brazil concluded that there was no association between these co-morbidities and acute kidney injury after undergoing cardiac surgery [20].

## Conclusions

The main predictor of poor renal outcome after cardiac surgery is preoperative creatinine, while other variables, such as age, gender, BMI, CPB time, diabetes, hypertension, and dyslipidemia did not show any risk to the renal outcome. These results deviate toward a trend that resembles recent literature; however, a prospective cohort with a larger sample size could show statistical significance in regard to CPB.
